# Phosphorus^☆^

**DOI:** 10.1016/j.advnut.2025.100555

**Published:** 2025-11-11

**Authors:** Mona S Calvo, Jaime Uribarri

**Affiliations:** Department of Medicine, Division of Nephrology, Icahn School of Medicine at Mount Sinai, New York City, NY, United States

## Phosphorus

**FIGURE 1:**
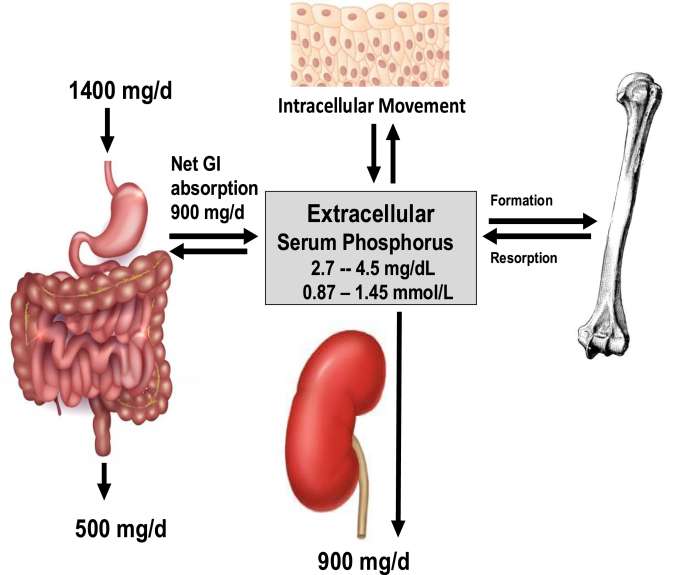
Basic elements in phosphorus homeostasis (net balance) with typical phosphate intake and normal kidney function.

**FIGURE 2:**
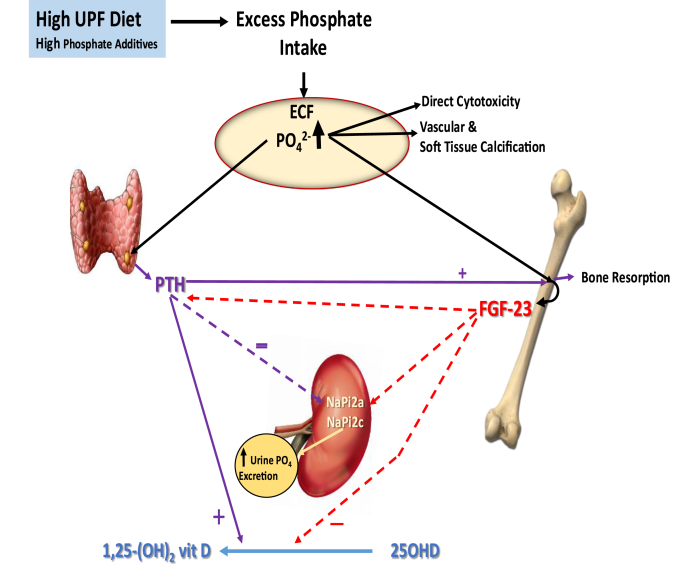
Disruption of complex hormonal regulation of phosphorus homeostasis with habitual high consumption of ultraprocessed food (UPF) containing phosphate additives. Stimulatory effects of excess phosphate intake and regulatory hormones PTH and FGF-23 are shown by solid lines and dashed lines showing inhibitory actions leading to decreased kidney function. Toxicity results from phosphate accumulation that promotes vascular and soft tissue calcification when kidney transporters reduce phosphate excretion.

Phosphorus, discovered in nature ∼350 y ago, is now considered a critically essential nutrient for all life forms functioning in metabolic pathways and structural cellular components that use the chemical properties of phosphate esters for energy, growth, reproduction, protein synthesis, and intracellular and extracellular structure. Because of extreme reactivity in nature, phosphorus occurs mainly bound to oxygen (phosphate anion) occurring as 2 forms of orthophosphates at the pH of the human body (H_2_PO_4_^−1^ and HPO_4_^−2^) forming inorganic phosphate salts. The nutrient occurs as organic phosphate when bound to carbon atoms of protein, lipids, nucleic acids, and other carbon compounds. Total body phosphorus content in a 70 kg man is ∼700 g with 85% in bones and teeth as calcium phosphate hydroxyapatite, 14% in soft tissues, and <1% in extracellular spaces. Unbound inorganic phosphate in the extracellular space is metabolically active and tightly maintained within a narrow serum concentration range (2.5–4.5 mg/dL) in adults [1]. The unbound phosphorus in circulation follows an inherent circadian rhythm over 24 h whose magnitude can fluctuate with dietary intake but with little to no change in the overall daily pattern. The form of phosphorus in the diet influences the mineral’s absorption, tissue distribution, and regulation of homeostasis. The kidney is a key organ in maintaining phosphorus balance (Figure 1) by keeping urinary losses the same as net gastrointestinal phosphorus absorption; normally, in the steady state, equal amounts of phosphorus are deposited and resorbed from bone daily. Current understanding of the homeostatic control of serum phosphorus within the normal range involves a complex bone-kidney-intestine network system operating through intricate, ordered multiple endocrine feedback loops between parathyroid hormone (PTH), fibroblast growth factor 23 (FGF-23), Klotho, and the active form of vitamin D (1,25-dihydroxy vitamin D). Figure 2 shows the endocrine response to high phosphorus intake and the potential for disease risk through soft tissue calcification with habitual dietary disruption of phosphorus balance [1]. Tissue-specific transport proteins respond to endocrine signals to increase or decrease phosphorus transport across cells of the small intestine, kidney proximal tubules, parathyroid glands, muscle, bone, and soft tissues [2]. It is increasingly apparent that such homeostatic control mechanisms maintaining phosphorus homeostasis can be disrupted by continuous excess phosphorus intake.

## Deficiencies

Phosphorus deficiency other than what occurs with excessive urinary losses of phosphate in in-born errors of metabolism or tumors is rare in the United States, likely because of the ubiquitous presence of phosphorus in plant and animal foods and to a greater extent in highly processed foods with phosphate additives. Hypophosphatemia is also described because of refeeding in anorectics or alcoholics.

## Diet Recommendations

In 1997, Dietary Recommended Intakes for phosphorus in adults, children, and infants shown in Table 1 were issued by the Institute of Medicine and to date remain unchanged [3]. Adult age-specific estimated average requirements (EARs) were derived using serum phosphate as a biomarker, whereas the biomarker in children was based on factorial estimates of accretion of phosphate into bone. The adult biomarker differs from those used in other countries and the use of serum phosphate remains very controversial. An intake of 580 mg phosphorus was estimated to meet the needs of 50% of the United States population; the adult EAR serves as the basis for determining the Recommended Dietary Allowance (RDA) for phosphorus, 700 mg, which is sufficient to meet the needs of 97.5% of the adult (≥19 y) population. Evidence of intakes far exceeding these dietary recommendations is also presented in Table 1 showing gender differences in mean usual phosphorus intakes from the most recent published national survey. Usual intakes are based on >1 survey measure to account for the day-to-day variability in intakes. For most adults and prepubescent children, usual phosphorus intake clearly exceeds the dietary guidelines, but not the Dietary Reference Intake measure used to assess the upper level (UL) of safe intake or UL [4]. The tolerable UL of safe intake or UL of 4000 mg/d of phosphorus for adults was also set in 1997; however, the method used to arrive at this value, which is almost 6 times higher than the RDA, is in question and suggests re-evaluation of the UL as adverse events with high phosphorus intake below the UL have been reported [1,5].

**TABLE 1:** Dietary Reference Intake (DRI) guidelines for phosphorus set by the Institute of Medicine(IOM) in 1997 [3] and mean usual daily intake for men and women in the NHANES 2017–March 2020 Prepandemic [4].

Age (y)	EAR (mg/d)	RDA (mg/d)	UL (mg/d)	Mean usual Pi intake (mg/d) (SE)	
				Men	Women
1–3	380	460	3000	1108 (31)	1011 (24)
4–8	405	500	3000	1252 (27)	1124 (20)
9–13	1055	1250	4000	1331 (46)	1291 (33)
14–18	580	1250	4000	1505 (49)	1064 (59)
19–30	580	700	4000	1501 (35)	1193 (23)
31–50	580	700	4000	1632 (38)	1198 (15)
51–70	580	700	4000	1583 (35)	1180 (20)
71+	580	700	4000	1463 (43)	1114 (22)

Abbreviations: EAR, estimated average requirement; RDA, recommended daily allowance; UL, tolerable upper intake level.

## Food Sources

Phosphorus is widely distributed in the food supply with organic, natural phosphorus being highest in dairy and meat categories and the less bioavailable phosphorus from plant-based foods in the form of phytate, notably whole grains. The 2 basic types of phosphorus in foods, natural and added or organic and inorganic, have different characteristics of absorption and influence the hormonal regulation of phosphorus balance. Natural or organic phosphorus is slowly and efficiently absorbed (40%–60%), whereas inorganic phosphorus salts added in processing are rapidly and efficiently absorbed (80%–100%) [6]. Excessive intake of phosphorus can also arise from the addition of phosphate additives in processing and the habitual pattern of foods consumed, particularly important for ultraprocessed food (UPF) consumption. The addition of phosphorus in industrial processing of foods significantly improves the shelf life, texture, taste, and convenience of many UPFs and are commonly used as excipients in many over-the-counter drugs (OTC) drugs [7]. The significant increase in food additive use in general [8] and specifically phosphate-based additives in UPF in the United States [9] has been suggested as contributing to the increased risk of chronic disease and mortality observed with high UPF consumption [7,10]. Use of phosphate additives contributes to the hidden phosphorus content of foods [11] making it impossible to determine the true nature of phosphorus consumption because the phosphorus content is not required on the label [9].

## Clinical Uses

Clinical use of phosphate dietary supplements to correct hypophosphatemia is largely limited to use in inherited disorders of phosphorus metabolism causing phosphate wasting and tumor-induced osteomalacia resulting in hypophosphatemia because of excessive tumor-secreted FGF-23 decreasing tubular phosphate reabsorption [1].

## Toxicity

Both extremes of dietary phosphorus deficiency and excess consumption can prove toxic. Currently, the main widely accepted clinical biomarker of phosphorus balance is serum phosphate. Thus, clinically, people may present with either hyperphosphatemia or hypophosphatemia, whose causes cannot always be attributed to dietary phosphorus intake. Hypophosphatemia, for example, may be a manifestation of urinary wasting of phosphorus because of inherited disorders of phosphorus metabolism and tumor-induced osteomalacia. In the United States, overconsumption is far more common than phosphorus deficiency and there is growing evidence of toxicity even in the absence of overt hyperphosphatemia [1]. Clinically, there are many disorders that may result from excess phosphorus intake, with or without overt hyperphosphatemia. Although this is an important concern for patients with chronic kidney disease (CKD), before and after dialysis, it is now becoming an issue of concern even for the general population. Epidemiological studies have shown an association between high dietary intake of phosphorus and mortality in the general population as well as significant links between dietary phosphorus excess and a variety of conditions such as hyperparathyroidism and bone health, cardiovascular disease, and progression of CKD. A role for hyperphosphatemia has also been postulated in accelerating senescence, poor cognitive function, and inducing cancer [1].

Many of the potential problems associated with excessive consumption of phosphorus can be more clearly understood in patients with progressive worsening of kidney function. Because fractional intestinal absorption remains unchanged at ∼60% in CKD, a usual intake of phosphorus in these patients will lead to phosphorus retention in the body with the subsequent increased risk of vascular calcifications, higher levels of PTH (less bone mass), and higher levels of FGF-23 (a cardiac toxin). It is important to understand that initially all these hormonal changes acting on the kidney may be able to maintain a normal serum phosphorus until late in CKD progression, but at the expense of increased metabolic disruption that will eventually lead to organ and tissue damage. From a nutritionist’s perspective, the single most important factor to realize is that dietary intervention should begin before serum phosphorus rises. Seminal work in dogs demonstrated that progressive proportionate reduction in phosphorus intake prevented the development of secondary hyperparathyroidism in a model of progressive CKD.

Some nutritional scientists misinterpret the laboratory dietary and clinical measurement of phosphorus that can affect dietary phosphorus research study design in animal models and humans [12]. Clinical measurement of phosphorus in biological fluids assesses only inorganic, not organic phosphorus. Furthermore, when a clinical laboratory reports a normal serum phosphate level of 4 mg/dL, it is expressed as inorganic phosphorus (phosphate anion) (molecular weight = 31); therefore, normal serum Pi = 4 mg/dL = 1.29 mmol/ L = 2.32 mEq/L at pH 7.4. Dietary phosphorus content is also expressed as phosphate, not elemental phosphorus, which needs to be considered in formulating experimental diets [1].

## Recent Research

As the use of food additives, including phosphate-containing additives, is one of the hallmarks defining UPF, dietary patterns high in processed and UPF are likely higher in bioavailable phosphorus, given their higher efficiency of absorption [7]. The use of phosphorus additives in processing markedly improves the shelf life, convenience of preparation, and perceived quality of many foods, and are widely present as excipients in frequently used prescription and OTC drugs, have global wide-spread use in processing food and beverages, and are believed to contribute to the addictive characteristics of highly processed packaged foods—all of which explain the increase in UPF consumption. The increased cumulative use of phosphate additives in food processing is hypothesized to result in dietary phosphorus intake greatly exceeding adult requirements and potentially, with time, disrupting the delicate balance in the tightly regulated serum phosphorus pool. This raises the issue of the potential toxicity of phosphorus when consumed in excess and urges further research into the role of phosphate additive use in food processing. It is important to emphasize that the adverse health outcome relationships between excess dietary intake and/or serum phosphate are only observed associations and not a proven cause and effect relationship. A recent small dietary intervention study focusing on limiting phosphate additives to improve outcomes in patients with CKD with and without dialysis fed phosphate additive–enhanced diets for 2 wk (∼2.8× RDA) followed by 6 wk of a low-phosphate additive diet (∼1.9× RDA) [13]. The authors reported that FGF-23, PTH, and urinary phosphorus were significantly lower after the low-phosphate additive diet in patients with moderate CKD. These findings , current clinical trials, and recent guidelines support the need for further research examining outcomes from longer exposure to high-phosphate additives and a clear definition of the UL of safe phosphorus intake in both patients with CKD and the general population [[14], [15], [16], [17]]. Currently, there is only 1 long-term, large, randomized controlled clinical trial in progress exploring whether lowering serum phosphate concentration using dietary phosphate binders in dialysis patients improves clinical outcomes [15].

A less-known aspect of the importance of phosphorus to global public health concerns its crucial role in sustaining the global food supply. Phosphate rock deposits in the earth’s crust are finite in number and content, not widely dispersed across countries, and are aggressively mined to supply industry with phosphate to produce chemical fertilizers. Phosphates are needed to meet the demands of modern global agriculture, animal feed, and the growing human population food supply [14]. As human populations urbanize, the ability to recirculate phosphorus back into food production and out of run-off and loss to environmental pollution has dwindled. Research to produce sustainable sources of phosphorus is critical to the global food supply and has now taken center stage and will continue to grow by enlisting the help of scientists with diverse backgrounds and skills [14].

## Author contributions

Both authors contributed equally.

## Funding

There was no funding section.

## Conflict of interest

JU reports financial support and administrative support were provided by Icahn School of Medicine at Mount Sinai, NYC, NY USA. MSC reports a relationship with Icahn School of Medicine at Mount Sinai NYC, that includes: speaking and lecture fees.
